# Parent SMART (Substance Misuse in Adolescents in Residential Treatment): Protocol of a Randomized Effectiveness Trial of a Technology-Assisted Parenting Intervention

**DOI:** 10.2196/35934

**Published:** 2022-02-28

**Authors:** Sara J Becker, Sarah A Helseth, Lourah M Kelly, Tim Janssen, Jennifer C Wolff, Anthony Spirito, Thomas Wright

**Affiliations:** 1 Department of Behavioral and Social Sciences Brown University School of Public Health Providence, RI United States; 2 Department of Psychiatry and Human Behavior Warren Alpert Medical School of Brown University Providence, RI United States; 3 Rosecrance Health Network University of Illinois College of Medicine Rockford, IL United States

**Keywords:** adolescent, residential, technology-assisted, substance use, parent, randomized controlled trial, RCT, intervention, eHealth, problem behaviour, problem behavior

## Abstract

**Background:**

Adolescents in residential substance use treatment are at extremely high risk for relapse following discharge to the community. Parenting practices, including parental monitoring and parent-adolescent communication, have been established as key predictors of adolescent substance use outcomes and relapse. However, traditional office-based therapy may not be feasible for parents who face structural and systemic barriers. There is a clear need for effective, accessible, and scalable interventions for parents of adolescents receiving residential substance use treatment. In a prior pilot randomized controlled trial, we tested Parent SMART (Substance Misuse among Adolescents in Residential Treatment)—a technology-assisted parenting intervention informed by extensive formative research—as an adjunct to residential treatment as usual (TAU). Parent SMART demonstrated high feasibility and acceptability, as well as evidence of effectiveness in improving parental monitoring and communication.

**Objective:**

This protocol paper describes a fully-powered randomized controlled pragmatic effectiveness trial of Parent SMART as an adjunct to residential TAU. We hypothesize that families who receive Parent SMART will demonstrate greater improvements in parenting skills, reductions in adolescent substance use, and reductions in adolescent problem behaviors relative to families that receive residential TAU. We will test the exploratory hypothesis that reductions in adolescent substance use will be partially mediated by improvements in parenting skills.

**Methods:**

Adolescent-parent dyads (n = 220 dyads; 440 total) will be randomized to either residential TAU only or Parent SMART+TAU. Parents randomized to Parent SMART will receive access to a networking forum, an off-the-shelf computer program called Parenting Wisely, and up to four telehealth coaching calls. Multimethod follow-up assessments consisting of self-reported parent and adolescent measures, a parent-adolescent in vivo interaction task, and 8-panel urine screens will be conducted 6, 12, and 24 weeks postdischarge from residential care. Measures will assess parenting skills, adolescent substance use, and adolescent problem behaviors. Analyses will be conducted using latent change score structural equation modeling.

**Results:**

The trial was funded in August 2021; ethics approval was obtained in August 2020, prior to funding. Due to concerns with the administrative interface in the pilot trial, the Parent SMART networking forum is currently being rebuilt by a different vendor. The programming is scheduled to be completed by December 2021, with recruitment beginning in February 2022.

**Conclusions:**

The proposed research has the potential to advance the field by serving a high-need, underserved population during a vital treatment juncture; targeting parenting practices (putative mediators) that have been shown to predict adolescent substance use outcomes; addressing barriers to accessing continuing care; and testing a highly scalable intervention model.

**Trial Registration:**

ClinicalTrials.gov NCT05169385; https://clinicaltrials.gov/ct2/show/NCT05169385

**International Registered Report Identifier (IRRID):**

PRR1-10.2196/35934

## Introduction

### Background

Adolescents with substance use disorders requiring a residential level of care typically present with severe symptoms and an array of co-occurring mental health, behavioral, motivational, legal, and environmental problems [1,2]. While a number of studies have found that residential treatment is associated with an acute reduction in symptoms and co-occurring problems [3,4], long-term outcomes of this population are far less encouraging [5]. Longitudinal studies indicate that the majority of adolescents will relapse within 90 days of discharge [6]. Such poor outcomes are likely at least partially attributable to the fact that only about 35%-45% of adolescents receive any continuing care [7,8].

To improve adolescent outcomes, it has been argued that continuing care interventions should move beyond solely including the adolescent to actively targeting parent engagement. Multiple systematic and meta-analytic reviews [9-12] have demonstrated that substance use interventions that include parents significantly outperform adolescent-only approaches. In recognition of this empirical support, the Residential Care Consortium released a white paper advocating that residential programs improve parent engagement, particularly prior to and following discharge to the community when disruptions in care are common [13]. Further research has suggested that adolescent substance use interventions targeting parental monitoring and communication, two key parenting processes that are protective against adolescent substance use [14,15], are associated with better adolescent outcomes than interventions targeting parent education only [16-18].

Building upon the extant research highlighting how positive parenting skills protect against adolescent substance use [19-21], as well as the research demonstrating the challenges with parent engagement in traditional office-based sessions [22,23], we developed a technology-assisted parenting intervention called Parent SMART (Substance Misuse among Adolescents in Residential Treatment). Parent SMART was designed as an adjunct to residential treatment for adolescents with substance-related problems. It consists of three elements—an online parent skills program, a parent networking forum, and telehealth coaching sessions. The online parenting skills program is an off-the-shelf product [24,25], whereas the networking forum and telehealth coaching sessions were specifically designed in response to formative research with parents of adolescents in residential treatment [26].

**Figure 1:**
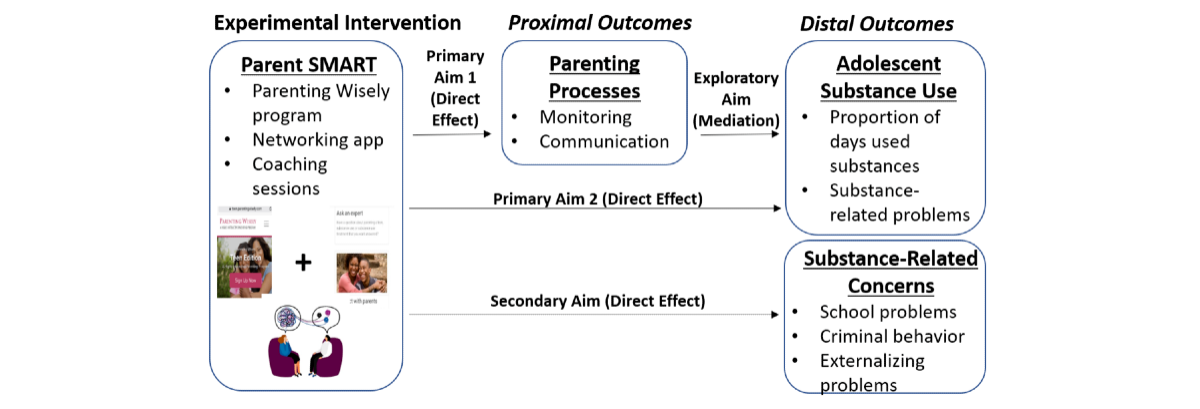
Overview of protocol aims. Parent SMART: Substance Misuse in Adolescents in Residential Treatment.

We previously tested the Parent SMART intervention via a pilot randomized controlled trial with 61 parent-adolescent dyads across two residential programs (one acute care and one standard residential care) [27]. Results of the pilot trial met or exceeded all of the recruitment, retention, acceptability, feasibility, and fidelity benchmarks established a priori as evidence that the intervention was worthy of further study in a larger trial. Parent SMART demonstrated excellent acceptability and feasibility, with parents who received Parent SMART reporting significantly higher levels of satisfaction and greater likelihood of recommending the services they had received than those who received residential treatment alone [27]. In addition, Parent SMART was associated with significant improvements in parental monitoring and communication, measured via both self-report scales and a behavioral intervention task, across both residential settings [28]. Among adolescents in the acute care setting, Parent SMART was also associated with significant reductions in days of binge drinking and school-related problems; we were underpowered to detect differences in the standard residential care setting, but a number of significant time effects were observed, revealing encouraging reductions in days of substance use, substance-related problems, and high-risk behaviors among adolescents in both conditions [27]. Building upon this prior work, the current protocol describes a fully-powered, randomized controlled trial testing the effectiveness of Parent SMART as an adjunct to residential TAU on parenting skills, adolescent substance use, and problems commonly related to substance use such as school truancy, externalizing behavior, sexual risk behavior, and criminal involvement [10]. A conceptual overview of the study’s specific aims is presented visually (Figure 1) and elaborated upon as follows.

### Specific Aims

**Primary aim 1:** Evaluate the effectiveness of Parent SMART+TAU versus TAU on proximal parenting outcomes. Hypothesis 1 is that, relative to those in TAU, parents receiving Parent SMART+TAU will exhibit greater improvements in parental monitoring and communication at 6, 12, and 24 weeks postdischarge from residential care.

**Primary aim 2:** Evaluate the effectiveness of Parent SMART+TAU versus TAU on distal adolescent substance outcomes. Hypothesis 2 is that, relative to those in TAU, adolescents whose parents receive Parent SMART+TAU will show greater reductions in the proportion of days substances were used outside a controlled environment and substance-related problems at 6, 12, and 24 weeks postdischarge from residential care.

**Secondary aim:** Evaluate the effectiveness of Parent SMART+TAU versus TAU on distal adolescent problem behavior outcomes commonly related to substance use: school problems, externalizing behaviors, sexual risk behavior, and criminal involvement. Hypothesis 3 is that adolescents whose parents receive Parent SMART+TAU will have greater reductions in a range of problem behaviors, including school truancy, externalizing behavior, risky sexual behavior, and criminal involvement at 6, 12, and 24 weeks postdischarge from residential, relative to adolescents in TAU.

**Exploratory aim**: Evaluate the extent to which change in parenting processes mediates change in adolescent substance outcomes. An exploratory hypothesis is that change in parenting processes (ie, communication, monitoring) at 6-weeks postdischarge from residential care will partially mediate primary adolescent substance use outcomes at 12 and 24 weeks.

## Methods

### Study Design and Ethical Considerations

Parent-adolescent dyads will be assigned to receive either residential TAU or Parent SMART+ residential TAU on a 1:1 schedule using a two-arm, parallel-group, randomized trial design. The Brown University Institutional Review Board has approved all study procedures described herein (ID# 2006002748). The study is registered in clinicaltrials.gov (NCT05169385). The timing of key study elements, including randomization and parent-adolescent assessments, is depicted in Table 1.

**Table 1:** Timing of key protocol elements.

		Residential treatment					Postdischarge period		
Timepoint		Enrollment	Baseline assessment	Allocation	Residential stay (2-45 Days)	6 weeks		12 weeks	24 weeks
**Enrollment**									
	Informed consent/assent	X							
	Masked allocation			X					
**Control condition**									
	Residential treatment as usual				X				
**Parent SMART condition**									
	Parenting Wisely program				X	X		X	
	Parent networking app				X	X		X	
	Telehealth coaching sessions				X	X			
**Assessment of primary aim 1**									
	Parental monitoring questionnaire		X			X		X	X
	Parental communication questionnaire		X			X		X	X
	Family assessment task		X			X		X	X
**Assessment of primary aim 2**									
	Proportion days used substances		X			X		X	X
	Substance-related problem scale		X			X		X	X
	Urine screens					X		X	X
**Assessment of secondary aim**									
	School-related problem scale		X			X		X	X
	Behavioral complexity scale		X			X		X	X
	Risky behavior scale		X			X		X	X
	Crime and violence scale		X			X		X	X

### Inclusion Criteria

A total of 440 participants—220 parent-adolescent dyads—will participate in this trial. Parent inclusion criteria are: (1) parent or legal guardian of adolescent aged 12-18 years admitted to residential treatment due to concerns about substance use; (2) will be primary guardian living with adolescent after their discharge from residential treatment; (3) fluent in English or Spanish; and (4) willing and able to complete a structured assessment prior to the adolescent’s discharge from residential treatment. Adolescents whose parents qualify are eligible to participate as long as they are willing to participate in the research and able to complete a structured assessment prior to their discharge from residential treatment.

### Recruitment

Parents will be referred to the study via a residential intake coordinator at the partner facility (see residential TAU), who will briefly inform parents about the study during the intake process. Interested parents will be given a consent to contact form on a study tablet to review and sign. The consent to contact form will provide a brief overview of the study and will collect the parent’s preferred contact information so that research staff can contact the parent at a later time. The research staff will then contact the parent to describe the study risks and benefits and complete a brief eligibility screener. Interested parents who qualify will provide informed consent to participate using electronic consent forms; parents of adolescent minors will also provide informed parental consent for their child to participate. The research staff will then work with staff at the residential facility to schedule a time to speak with the adolescent via phone or Zoom to describe the study and to complete a brief screener. Interested adolescents will provide informed assent (for those age 13-17 years) or informed consent (for those age 18 years) via electronic forms. Parents and adolescents must independently provide consent and assent, respectively, for an adolescent-parent dyad to enroll in the study.

### Anticipated Participant Demographics

Based on the participants enrolled in the pilot trial [27], parents are expected to be predominantly biological mothers (about 80%), with the remainder comprised of biological fathers, adopted parents, and other legal guardians. For adolescent characteristics, we expect approximately 10% of adolescents to identify their gender as nonbinary, with the remainder identifying as male (45%) or female (45%). We anticipate about 25% of adolescents will identify as Hispanic. We expect adolescents to self-identify in the following racial categories: White (65%), Black or African-American (12%), Multiracial (15%), and Asian or Asian American (2%). We expect about 6% of adolescents to select “prefer not to answer” as their preferred race.

### Randomization

Parent-adolescent dyads will be randomized immediately after completing the baseline assessment using the urn randomization method [29], which systematically biases randomization in favor of balance across conditions. Dyad randomization will be balanced according to adolescent biological sex at birth (male vs female), planned length of stay in residential (0-10 days vs 10+ days), and parent’s preferred language (English vs Spanish). Random assignments will be made using an Excel urn generator developed by Stout and colleagues for Project MATCH [22]. Parents will be informed about their treatment assignment shortly after randomization. Residential staff and study research staff conducting follow-up assessments will be blind to the condition.

### Interventions

#### Residential TAU

TAU consists of the standard residential services offered to adolescents at our partner facility and will be received by families in both conditions. The partner residential treatment program, Rosecrance Health Network, offers residential treatment for adolescents at two locations, one in Rockville, Illinois, and one in Sioux City, Iowa. Length of stay ranges from 2 to 45 days, depending on the adolescent’s level of need and insurance coverage. Adolescents at Rosecrance Health Network receive about 15 hours of educational programming and 25 hours of treatment per week in a predominantly group-therapy model based on dialectical behavioral therapy [30]. Each adolescent is also assigned a primary counselor who checks in with them at least twice per week. Medication management is offered by a nurse practitioner or psychiatrist as needed. Prior to discharge, parents receive standard discharge planning, which consists of a customized relapse prevention plan as well as either a referral to a new outpatient therapy provider or a return to a prior outpatient therapy provider. Families receive referrals to a psychiatrist or local self-help group as indicated. Postdischarge, families are encouraged to phone the intake coordinator for further referrals or support.

Following randomization, parents will be given an educational pamphlet about adolescent substance use, parenting skills, and local community resources, consistent with usual resource provision at the partner site.

#### Parent SMART

Parents randomized to Parent SMART will receive a technology-assisted intervention containing three core elements, which were packaged in response to formative feedback described extensively [26] and tested in the pilot trial [27,28]. All three elements are available in both English and Spanish.

##### Online Parent Skills Program

**Figure 2:**
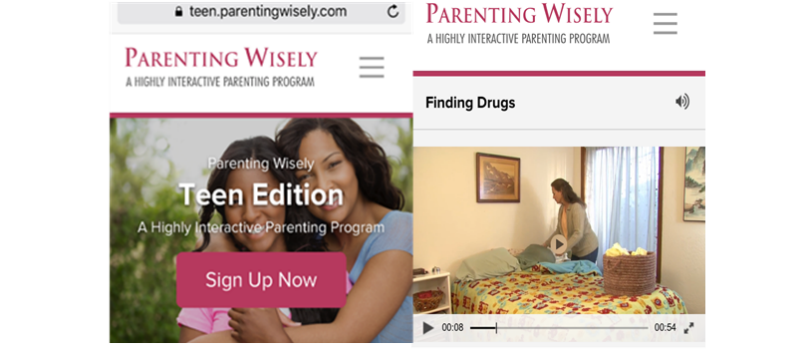
Parenting Wisely computer program.

Parents will receive a 24-week subscription to an off-the-shelf, online parent skills program called Parenting Wisely (Figure 2) shortly after randomization to Parent SMART. Parenting Wisely (Ser Padres Con Sabiduría) is a self-administered, multimedia online program that has demonstrated effectiveness in reducing youth problem behavior across multiple independent studies [24,31-33]. The program contains nine modules, each of which corresponds with a common family problem (eg, finding drugs, monitoring of friends, sibling conflict, etc). Core skills emphasized across the Parenting Wisely modules include two communication skills (I-Statements and Reflective Listening) and two monitoring skills (Contracting and Asking Key Questions). The program also comes with a workbook that outlines all the vignettes, along with a glossary of terms, sample behavior charts, and practice exercises.

After selecting a module, parents are shown a video clip depicting a family struggling with that problem. Parents then select from three possible video vignette solutions, only one of which demonstrates an effective approach. Parents then receive feedback via video enactment of their chosen solution. The video enactment labels specific parenting skills, explains why the solution was either effective or ineffective and discusses how effective parenting skills (eg, monitoring and communication) can protect against common family problems.

The full Parenting Wisely program takes 3-5 hours to complete depending on user speed and depth of use. Parents will have a unique login, which will allow the research team to monitor their usage. In the pilot trial, 83% of the parents randomized to Parent SMART completed at least two modules, and 33% completed at least five modules. Overall, parents completed a mean of 3.8 modules (SD 2.3, range 1-9 completed modules) [28].

##### Networking Forum

**Figure 3:**
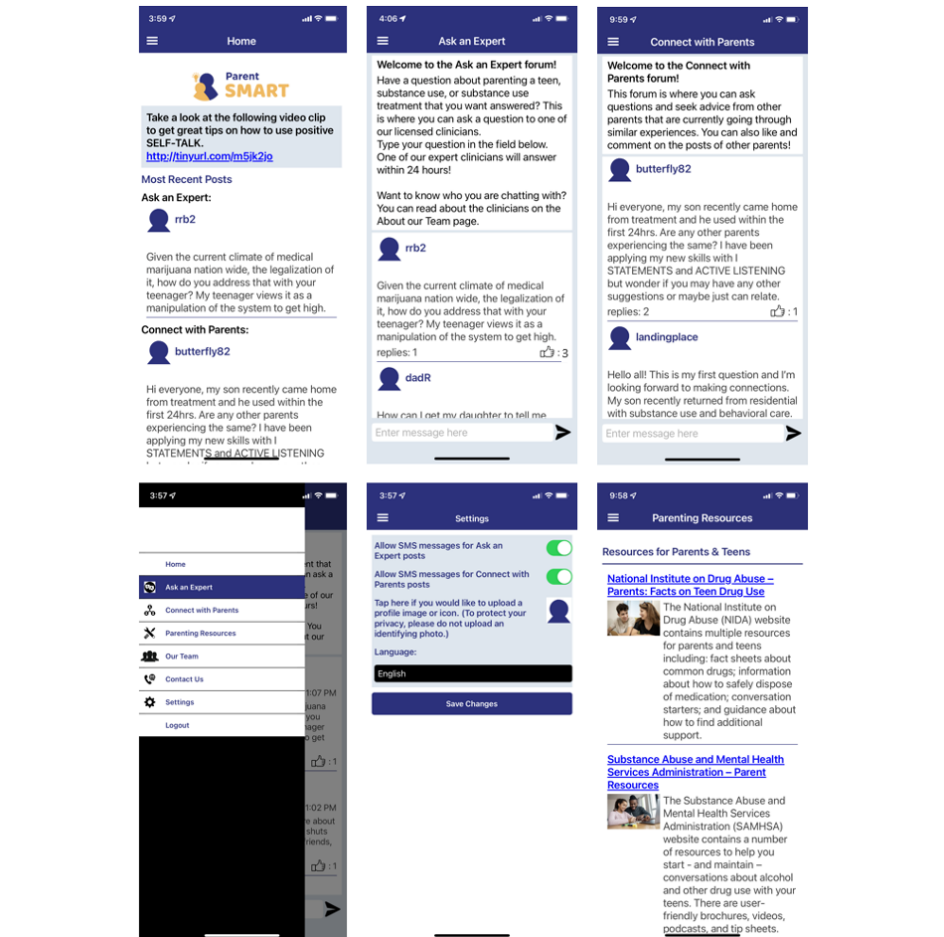
Parent SMART (Substance Misuse in Adolescents in Residential Treatment) app interface.

Parents will also receive access to a networking forum containing two main channels (Ask an Expert and Connect with Parents) along with parenting resources, information about the study team, and customizable settings (Figure 3). The Ask an Expert channel allows parents to pose questions to a team of licensed clinical psychologists, whereas the Connect with Parents channel enables parents to pose questions and comments to other parents of adolescents in residential treatment. The networking forum will be accessible via smartphone app or web browser. The smartphone app will use push notifications to send a daily “Tip of the Day!” offering encouragement, reminders to use the forum, and/or links to Parenting Wisely videos; if the push notification is not opened, parents will receive the notification via SMS message two hours later. In the settings tab, parents can also subscribe to SMS notifications every time someone posts in one of the two main channels.

The networking forum has been carefully designed so that parents can view all content (including push notifications and SMS messages) in either English or Spanish, using a mix of static content that is pretranslated (eg, page headings and team bios) and user-generated content that is translated via a Google translator set-in. Parents will select their preferred language in the forum’s user settings. A bilingual expert moderator will review and correct translation errors. This will allow Spanish-speaking parents to interact with posts by English-speaking parents in both forums and vice versa with minimal disruption. On an ongoing basis, the expert moderator will review all posts submitted to both channels to remove any potentially identifiable information or objectionable material.

Posts submitted to the Ask an Expert forum will be answered by a team of expert adolescent clinical psychologists to ensure continuous coverage. Responses to parent questions will follow a specific formula: validation of concern, reference to a specific Parenting Wisely core skill, and reference to a specific Parenting Wisely module(s) most relevant to the concern. By contrast, posts submitted to Connect with Parents will be viewed by other parents, who can “like” posts or provide comments.

In the pilot trial, 21 of the 30 parents (70%) randomized to Parent SMART posted a total of 16 times in Ask an Expert and 50 times in Connect with Parents. Qualitative analysis of the posts revealed five key themes: parenting skills, parent support, managing the transition home from residential care, adolescent substance use, and family functioning [34]. Parents will be told to expect replies within 24 hours; the average lag time in our pilot trial was 1 to 2 hours.

##### Coaching Sessions

Telehealth coaching sessions will be offered to support the customization of the content in the Parenting Wisely computer program and encourage engagement in the networking forum. Each session will consist of the rationale for a specific parenting skill, review of a Parenting Wisely module, practice applying the skill to a specific problem, and consideration of questions that the parent would like answered via the networking app. Focal parenting skills covered will align exactly with the Parenting Wisely skills to address parental monitoring (I-Statements and Reflective Listening) and communication (Contracting and Asking Key Questions). Parents will be asked to schedule sessions at times when they can access the internet to view Parenting Wisely modules. The initial 60-minute session will consist of an orientation to the Parenting Wisely program and networking app. Subsequent 45-minute sessions will follow the same basic outline without the orientation. When parents have questions or introduce problems/ concerns about their teen, the coach will first provide validation and then discuss how the parents’ problems can be addressed using a specific Parenting Wisely core skill. For homework, parents will be encouraged to complete Parenting Wisely modules that map onto their unique concerns (with reference to specific pages of the parent workbook for additional practice) and to use the networking forum to solicit feedback from either an expert or another parent.

In the pilot trial, 29 of the 30 parents randomized to Parent SMART initiated coaching sessions. On average, parents attended 2.7 coaching sessions, out of a maximum of 4 (SD 1.1, range 1-4) [27].

#### Parent SMART Coach Training, Fidelity and Competence

Parent SMART sessions will be delivered by BA-level or MA-level coaches, consistent with the approach used in the pilot trial. At least one counselor fluent in Spanish will be available at all times. A licensed clinical psychologist (protocol Principal Investigator), with expertise in training front-line community counselors, developed the training materials and will lead an initial 2-hour training. Prior to the training, coaches will be asked to review the Parent SMART manual, complete two modules in the online Parenting Wisely program, visit the parenting networking forum, and listen to two prerecorded coaching sessions that demonstrate all session elements. During the training session, coaches will conduct multiple role plays of the sessions to gain practice delivering the intervention content.

Before being assigned Parent SMART+TAU participants, each coach must submit two audio-recorded role plays that meet adherence and competence benchmarks. Counselor adherence to each Parent SMART session will be rated via study-specific adherence checklists that range from 0 (no elements covered) to 15 (all elements covered), with the target level of adherence set at 80% coverage. Competency will be rated via the well-validated 6-item “General Therapeutic Skills” subscale of the Cognitive Therapy Rating Scale [35,36]. Items will be scored on 6-point Likert scales ranging from 1 to 6 and then averaged; a mean score ≥4 will indicate competence. All sessions will be rated for adherence and competence by research staff trained in the rating process, with at least 25% of sessions double coded. A different licensed clinical psychologist (second author) will lead weekly supervision meetings with coaches, focused on tailoring sessions to address each family's unique presenting concerns.

A total of 78 sessions occurred in the pilot trial: all were rated on the coach’s adherence to protocol and competence by a single coder, and 33% were double coded. In total, 87% of sessions met the adherence, and 100% met the competence benchmark [27]. Agreement between coders was excellent: inter-rater reliability measured via the intraclass correlation coefficient was 0.92 for adherence and 0.86 for competence.

### Study Assessments

Parents and adolescents will complete a baseline assessment shortly after admission to the partner facility. Follow-ups will be conducted by research staff blind to condition at 6, 12, and 24 weeks postdischarge. The baseline assessment will be approximately 90-120 minutes for adolescents and 45-60 minutes for parents; each follow-up assessment will take approximately 60 minutes for adolescents and 30 minutes for parents. If a parent loses physical custody of the adolescent postdischarge, adolescent data will still be collected, and parent data will be classified as missing; administration of the full parent battery will be resumed if the parent regains physical custody. If an adolescent turns 18 over the course of the 24-week follow-up period, then the adolescent will be reconsented as an adult at the next relevant follow-up visit.

#### Primary Aim 1: Parenting

To address primary aim 1, we will conduct a multimodal assessment of parental monitoring and parental communication. Specifically, we will use parent and adolescent report measures with strong psychometric properties, as well as an in vivo family assessment task. Prior research suggests that adolescent report of parent behavior is more predictive of adolescent risk behavior than parents’ self-reported behavior [14]. As such, adolescent reports will be used to capture parent behaviors, whereas parent self-report will be used to measure adolescent behaviors.

##### Parent Monitoring Questionnaire

The Parental Monitoring Questionnaire [37] is a 24-item youth and parent-reported measure containing three subscales. We will use the following versions of the subscales to assess dimensions of parental monitoring: child disclosure (parent report), parent solicitation (adolescent report), and parental control (adolescent report). Subscales were reliable in our pilot trial (α=.76-.87) and have been shown to correlate with adolescent mental health problems, deviant peer relationships, and family discord [37].

##### Parent-Adolescent Communication Scale

The Parent-Adolescent Communication Scale [38] is an adolescent and parent reported measure with two subscales. The adolescent-reported version only will be used to assess positive (general family communication) and negative (problems with family communication) aspects of parent-adolescent communication. Subscales had good reliability (α=.71-.92) in our pilot [28] and have been shown to correlate with adolescent engagement in risk behavior [38].

##### Family Assessment Task

The family assessment task (FAsTask) is an audio-recorded family problem-solving task that will be used to provide an in vivo assessment of parenting skills. Three tasks (5 minutes each) will assess parental monitoring and communication: (1) limit setting: parents lead a discussion about a time they had to set a limit); (2) substance use norms: parents lead a discussion on family views about substance use; and (3) monitoring and listening: adolescent leads a discussion about a time they were unsupervised with their peers and parents react. Each item will be rated on a 9-point Likert scale by trained research staff blind to condition using a structured codebook initially developed by Dishion and colleagues [39] and adapted by the research team. Ratings between 1 and 3 will indicate poor parenting skills, ratings >3 to <6 will indicate average parenting skills, and ratings of 6 and above will indicate strong parenting skills. Individual ratings will yield five distinct scale scores: limit setting (10 items), parent substance use beliefs (4 items), parent substance use communication (6 items), adolescent disclosure (7 items), and parental monitoring (9 items).

In the pilot trial, 20% of FAsTasks were double coded with an excellent mean inter-rater reliability of 94%. Moreover, the FAsTask was sensitive to change, with all five subscales demonstrating significant time by treatment condition interactions, favoring Parent SMART+TAU relative to the TAU-only condition in our pilot trial [28].

#### Primary Aim 2: Substance Use

Adolescent substance use will be assessed via the Global Appraisal of Individual Needs-Core (GAIN) [40], a well-validated, structured, comprehensive interview. The GAIN has intake and follow-up versions, which each contain over 100 analyzable scales across eight sections (background, substance use, mental health, physical health, risk behaviors, legal, vocational, and environmental). We do not obtain corroboration from parents or peers since parents often underestimate adolescent use [41-43] and since peer corroboration raises issues of confidentiality and unintentional harm [44]. We will assure adolescents of confidentiality, as self-reported substance use has shown reliability when confidentiality is assured [45].

##### Proportion of Days Substances Used Outside a Controlled Environment (Past 90 Days)

A series of GAIN items will evaluate days of substance use over the past 90 days, as well as days the adolescent was in a controlled environment (eg, residential treatment or justice facility). This will allow calculation of the proportion of days that the adolescent was outside of a controlled environment on which substances were used. The GAIN days of use items have shown excellent comparability [46] to the well-validated Timeline Follow-Back Interview [47,48]. Like the Timeline Follow-back, the GAIN will assess days of alcohol and drug use using a calendar with temporal cues (eg, holidays and special events) to facilitate recall. The proportion of days all substances are used and separate days of binge drinking, marijuana, all other drugs, stimulant, and opioid use will be examined.

##### Substance-Related Problems Scale

The GAIN substance-related problem scale will be used to obtain a count of substance use symptoms experienced over the past 90 days in line with the latest diagnostic criteria for a substance use disorder [49]. Diagnoses made from this scale have demonstrated good test-retest reliability (κ=.55) [50] and accurately predict independent, blind staff ratings of the presence of substance use disorder (κ=.91) [51]. Reliability in our pilot trial (α=.78) [27] was consistent with prior studies using the same items (α=.76) [51].

##### Urine Drug Screens

Urine drug screens will be used to corroborate self-reported abstinence and gauge under-reporting of substance use in adolescents. Urine screens will be collected via 8-panel dip urine screens at the 12-month and 24-month follow assessments (75% testing rate in our pilot trial). Samples will be tested for metabolites of marijuana, cocaine, amphetamines, methamphetamines, barbiturates, phencyclidine, opiates, and benzodiazepines.

#### Secondary Aim: Adolescent Problem Behavior

GAIN scales will be used to assess common behaviors often linked to adolescent substance use [52-55]; higher scores indicate more problems. Consistent with the substance use scales, these scales will use a 90-day recall. Of note, in the pilot trial, we used an abbreviated version of the GAIN that contained briefer versions of some of these scales; thus, we report psychometric data from the measure validation data trial.

##### Behavioral Complexity Scale

This scale counts 33 externalizing behaviors, including symptoms of conduct, inattention, and hyperactivity. The full scale has good test reliability for total symptoms (*r*s=.7-.8) and strong internal consistency (α=.94) [40].

##### School-Related Problems

This four-item scale counts school-related problems, including truancy, chronic tardiness, poor grades, and cutting class. The full scale has demonstrated significant associations with adolescent employment problems, substance use, and cumulative stress in prior studies [56].

##### Risky Sexual Behavior

This three-item scale evaluates the number of risky sex-related behaviors, including sexual activity with multiple partners, unprotected sex, and sex while under the influence of alcohol or drugs. The full scale has demonstrated concordance with adolescent substance use and mental health problems in prior studies [40].

##### General Crime Scale

This 31-item scale assesses the number of behaviors the adolescent engaged in indicative of general conflict, as well as property, interpersonal, and drug crime. Test-retest reliability of the full scale has been shown to be excellent with strong internal consistency (α=.90) [40].

##### Covariates: Demographics

Parent and adolescent sociodemographics will be assessed and controlled for as covariates. Parents will complete a routine sociodemographic form. Adolescents will complete the GAIN Background section, which collects routine sociodemographic variables including age, sex, gender identity, ethnicity, race, and grade in school. Items in other GAIN sections indicate the adolescent’s history of substance use or mental health treatment utilization, data that will be used to control for treatment received.

### Data Analysis Plan

#### Overview

The data analysis plan contains periodic data quality checks, early generation of analysis variables, and mock study table generation to provide a check on completeness of study data. The study statistician will be blind to treatment conditions and follow a protocol established a priori.

Hypotheses will be tested with a latent change score modeling [57] approach estimated within a structural equation modeling framework [58]. The structural equation modeling framework allows direct estimating and testing hypotheses of interest, including treatment effects and mechanisms of action across multiple outcome variables. Compared to latent growth modeling, latent change score modeling is flexible (ie, not structured) with respect to the shape of the trajectory of change. This approach avoids misspecification of the change trajectory and allows for the testing of mediation hypotheses (exploratory aim). As a mixed repeated measures approach based on maximum likelihood estimation, the model produces unbiased effect estimates and standard errors under the missing at random assumption. The missing at random assumption is less restrictive than the missing completely at random assumption that is implicit in complete case or carry-forward methods for missing data and consistent with the intent-to-treat principle [59]. We will focus on intent-to-treat analyses to optimize rigor and account for the fact that families randomized to Parent SMART may engage in passive activities, such as lurking in the app or reading push notifications. Supplementary completer analyses will assess effects among those who complete at least two telehealth coaching sessions and two Parenting Wisely modules. Analyses will also control for contact time (eg, number of modules completed, number of posts in the app, and number of sessions attended) as a covariate in analyses of primary and secondary outcomes. The full analysis plan will be coded and executed on collected data at the end of Year 1 to identify errors so final analyses can be completed quickly.

Primary aims 1 and 2 and the secondary aim compare trajectories of change using data from baseline, 6, 12, and 24 weeks follow-up on parenting processes, adolescent substance use, and adolescent problems, respectively. These aims will be evaluated in latent change score models with separate change effects for each of the adjacent time points [57]. For each aim (primary aims 1-2 and secondary aim), multiple outcomes are specified. For example, primary aim 1 specifies parental communication (two subscales) and parental monitoring (three subscales). Primary aim 2 includes days of substance use as well as problems related to substance use. A single latent variable at each time point will be used to define a composite outcome reflecting shared covariance among the multiple outcome indicators. Measurement invariance will be tested over time, and noninvariance will be assumed if necessary. “Point-in-time analyses” will be conducted to assess preliminary effects at 6 weeks and then at 12 weeks.

The exploratory aim examining potential mediators will be evaluated by bringing outcome models together in a single multivariate model. The exploratory hypothesis posits that positive changes in parenting processes at 6 weeks will be related to reductions in adolescent substance use and adolescent problems at 12 and 24 weeks. A bivariate change score model will evaluate change in parenting processes and change in adolescent substance use and related problems simultaneously and introduce a residual covariance term describing the association of change in parenting and change in adolescent outcomes. Time lagged regression coefficients will then be applied to test whether a change in parenting processes at 6 weeks leads to lower adolescent substance use and adolescent problems at 12 and 24 weeks. Examples of such an approach to evaluating mediation and moderation effects in the context of randomized trials can be found in McArdle and Prindle [60].

#### Power

The study’s primary and secondary aims were specifically powered to detect small to medium effect sizes on the Time*Condition interactions, based on: (1) the effect sizes detected in our pilot trial, which were small to medium in size, and which matched the proposed population in terms of severity; and (2) the effect sizes of the online parenting program on parenting processes detected in prior clinical trials, which were medium to large [24,32,33]. With a sample size of 220 dyads, we will have 80% power to detect a 0.38 standard deviation effect size or larger with a type I error risk of .05.

## Results

This pragmatic effectiveness trial received notice of grant award on August 1, 2021. Ethics approval was obtained a full year prior to funding, in August 2020. Due to concerns with the administrative interface in the pilot trial surfaced by the study investigators, the Parent SMART networking app is currently being rebuilt by a new vendor, Mooseworks Software LLC. The programming has been fully completed as of December 22, 2021, and demonstrations are scheduled with the recruitment partner in January 2022. Recruitment is scheduled to commence in February 2022.

## Discussion

### Summary

Given the poor long-term outcomes of adolescents in residential care [1,2,51], strategies to improve adolescent substance use treatment outcomes following discharge are sorely needed. This pragmatic effectiveness trial will compare residential TAU to a multicomponent technology-assisted parenting intervention that has shown strong evidence of feasibility and acceptability [27] as well as preliminary effectiveness in improving parent-adolescent communication and parental monitoring. This trial will specifically address calls for improvements to residential care for adolescents put forth by the Residential Care Consortium [13] by promoting the engagement of parents during the transition from residential treatment to continuing care.

### Potential Strengths and Limitations

This pragmatic effectiveness trial is characterized by a number of strengths. Most notably, Parent SMART is a novel technology-assisted intervention that was developed based on extensive formative research with parents, adolescents, and residential treatment staff and is supported by encouraging data from a rigorous pilot randomized controlled trial. This study will build upon the successful pilot trial by conducting a fully-powered evaluation of the intervention’s effects on parental monitoring, parent-adolescent communication, adolescent substance use, and adolescent behavioral outcomes. Moreover, this study will move beyond simply testing the effectiveness of the intervention by specifying and examining putative mediators of change. Finally, the Parent SMART intervention is highly scalable: it augments an off-the-shelf intervention with a parent networking app and telehealth sessions that require limited training to deliver. Future work will seek to test different delivery models of Parent SMART to support long-term sustainability: (1) delivery by trained residential staff who bill for services, and (2) delivery by outside vendors, purchased as part of a technology package paid for by the residential center as part of the bundled rate.

In addition to these strengths, several limitations should be considered. A key limitation is that the Parent SMART+TAU and TAU conditions do not offer an equivalent dose of contact time. As a result, favorable results associated with Parent SMART may be attributable to a higher overall dose of treatment. This decision was made because our goal was to assess whether the package intervention was a feasible, acceptable, and effective adjunct to residential services delivered under real-world conditions. A related limitation is that Parent SMART is a bundled, multicomponent intervention, which will limit the ability to determine which aspects of the intervention (ie, the computer program, parent networking app, telehealth sessions) are driving observed effects. To mitigate these limitations, we will carefully control for each type of contact time (eg, number of telehealth sessions completed, time spent in the app, number of parenting skills modules completed) in analyses of primary and secondary outcomes.

### Conclusions

If found to be effective, Parent SMART could offer a relatively low-cost, highly scalable adjunctive strategy to engage parents of adolescents receiving residential treatment. Layering in an adjunctive technology-assisted intervention could potentially extend the reach of residential TAU and promote engagement in continuing care during a time when adolescents are at especially high risk of relapse. Parent SMART could offer highly significant public health benefits by improving vital parenting processes shown to protect against substance use and substance-related problems during the vulnerable transition from residential care to the community. Future investigations should seek to evaluate the feasibility, acceptability, and preliminary effectiveness of Parent SMART intervention in settings where adolescents receive comparable stabilization and support, such as partial hospital and intensive outpatient programs.

## Appendix

Multimedia Appendix 1 Peer review reports.

## Abbreviations

FAsTask: Family Assessment Task
GAIN: Global Appraisal of Individual Needs
Parent SMART: Substance Misuse among Adolescents in Residential Treatment
TAU: treatment as usual
