# Supplementary material for: Parent SMART (Substance Misuse in Adolescents in Residential Treatment): Protocol of a Randomized Effectiveness Trial of a Technology-Assisted Parenting Intervention
Source: JMIR Res Protoc. 2022 Feb 28;11(2):e35934. doi: 10.2196/35934 (PMC8922142; doi:10.2196/35934)
Supplement: Multimedia Appendix 1 [file resprot_v11i2e35934_app1.pdf]

SUMMARY STATEMENT

(Privileged Communication)

PROGRAM CONTACT:
CARRIE Mulford
301-827-6473
carrie.mulford@nih.gov

Release Date: 06/17/2020
Revised Date:

Application Number: 1 R01 DA052918-01

Principal Investigator

BECKER, SARA J.

Applicant Organization: BROWN UNIVERSITY

Review Group: IPTA
Interventions to Prevent and Treat Addictions Study Section

Meeting Date: 06/04/2020
Council: OCT 2020
Requested Start: 12/01/2020

RFA/PA: PAR19-212
PCC: CM/CFM
Dual IC(s): AA

Project Title: Improving Outcomes of Adolescents in Residential Substance use Treatment via a Technology-Assisted Parenting Intervention
SRG Action: Impact Score:23 Percentile:3
Next Steps: Visit https://grants.nih.gov/grants/next_steps.htm
Human Subjects: 30-Human subjects involved - Certified, no SRG concerns
Animal Subjects: 10-No live vertebrate animals involved for competing appl.
Gender: 1A-Both genders, scientifically acceptable
Minority: 1A-Minorities and non-minorities, scientifically acceptable
Age: 6A-Children and Adults, scientifically acceptable

Project Year	Direct Costs Requested	Estimated Total Cost
1	499,659	807,441
2	499,613	807,367
3	499,827	807,713
4	499,932	807,882
5	499,986	807,969
TOTAL	2,499,017	4,038,372

ADMINISTRATIVE BUDGET NOTE: The budget shown is the requested budget and has not been adjusted to reflect any recommendations made by reviewers. If an award is planned, the costs will be calculated by Institute grants management staff based on the recommendations outlined below in the COMMITTEE BUDGET RECOMMENDATIONS section.

1R01DA052918-01 Becker, Sara

RESUME AND SUMMARY OF DISCUSSION: This application proposes a randomized controlled trial to test a technology-assisted parenting intervention to prevent relapse among adolescents in short-term residential substance use treatment. During the discussion, reviewers were enthusiastic about the potential to improve outcomes in this difficult-to treat population with high relapse rates by engaging parents in treatment. Reviewers were particularly impressed by the strong rigor of the prior research supporting the intervention's preliminary effectiveness, acceptability, parent satisfaction, and feasibility based on the investigators' R34 pilot study. The rich data from that study, which piloted virtually all aspects of the proposed study, along with the investigators' responsiveness to feedback from that study, allowed the investigators to make well-informed decisions, resulting in a rigorous, well-conceived approach. The investigative team is exceptional, with a long history of collaboration. The primary concern expressed by reviewers centered around the scalability of an intervention that relies heavily on ongoing staff support and that may be difficult to integrate into regular practice. A concern was also raised about compliance with the long assessments. Overall, however, reviewers agreed that the strengths of this application outweigh its weaknesses and the project is likely to have high impact.

DESCRIPTION (provided by applicant): Adolescents in residential substance use (SU) treatment have the most serious SU disorders and the highest rates of psychological, behavioral, legal, environmental, and vocational problems. Adolescents in residential SU treatment are also at high risk of relapse, with follow-up studies demonstrating that 60% of discharged adolescents relapse within 90 days. Parenting practices, including parental monitoring and parent-adolescent communication, have been established as key predictors of adolescent SU outcomes and likelihood of relapse, but parents are notoriously difficult to engage in adolescent SU treatment. Accordingly, there is a clear need for effective, accessible, and scalable interventions for parents of adolescents receiving residential SU treatment. Building upon our successful NIDA-funded R34, this study evaluates a technology-assisted parenting intervention called Parent SMART (Substance Misuse among Adolescents in Residential Treatment), which has evidence of high feasibility and acceptability, as well as preliminary evidence of effectiveness, as an adjunct to short-term residential treatment as usual (TAU). Parent SMART centers around an off-the-shelf computerized intervention, Parenting Wisely (PW), which has demonstrated robust evidence of improving parenting skills and reducing youth behavior problems in multiple clinical trials. We conducted extensive formative research with parents, adolescents, and residential staff to guide the development and delivery of two highly scalable enhancements: 1) access to a state-of-the-art mobile networking app; and 2) up to four telehealth coaching sessions to tailor PW content. Our networking app allows parents to submit questions to an SU expert and connect with other parents of adolescents in residential SU treatment in real-time, while reinforcing parenting skills via "Tip of the Day!" push notifications. In our pilot trial, Parent SMART was highly feasible and acceptable, and demonstrated evidence of effectiveness in improving parental monitoring and communication, reducing days of adolescent binge drinking and all other drug use, and reducing school-related problems, among parents in short-term residential treatment. This R01 proposes a fully powered evaluation of Parent SMART. Adolescent-parent dyads (n = 220 dyads; 440 in total) will be randomized to receive either TAU only or Parent SMART + TAU. Multi-method follow-up assessments (i.e., self-report parent and adolescent measures, parent-adolescent in vivo interaction task, 8-panel urine screens) will be conducted 6-, 12-, and 24-weeks post-discharge, to examine parenting skills, adolescent SU, and adolescent problem behaviors. Exploratory analyses will test whether improvements in parenting skills partially mediate reductions in adolescent SU. The proposed research has the potential to advance the field by: serving a high-need, underserved population during a vital treatment juncture; targeting parenting practices (putative mediators) that have been shown to predict adolescent SU outcomes; addressing barriers to accessing continuing care; and testing a highly scalable intervention model informed by extensive formative research.

PUBLIC HEALTH RELEVANCE: Adolescents in residential substance use treatment have high risk of relapse after discharge, but it is extremely difficult to engage these adolescents and their parents in continuing care. This study aims to improve the outcomes of adolescents following discharge from residential substance use treatment by offering their parents a novel technology-assisted intervention. Technology-assisted interventions have the potential for marked public health impact by extending the reach, duration, and scalability of evidence-based care.

CRITIQUE 1

Significance: 3
Investigator(s): 1
Innovation: 3
Approach: 3
Environment: 1

Overall Impact: Parents play a key role in adolescent relapse prevention, but they have been difficult to engage in treatment. This R01 application seeks to test a packaged parenting technology assisted intervention to prevent relapse among adolescents in two short-term residential programs with 220 parent/adolescent dyads who will be randomly assigned to the intervention or usual care. The intervention package includes an off-the-shelf parenting app, telehealth sessions (up to 4), and a networking app through which parents can connect with clinicians or other parents. Score driving factors were the potential of the intervention to engage parents with minimal burden to staff; the rigor of the pilot and formative work, in which virtually all aspects of the study have been piloted; and the responsiveness to feedback from the pilot (e.g., targeting short-term residential programs based on better effectiveness relative to long-term programs; the timing of intervention delivery). Also important were their strategies in place to recruit and retain underserved ethnic minority families. Concerns pertained to whether components of the intervention would be able to be scaled up, in particular, the research team is responsible for moderating the networking app, which causes concerns about external validity. Also, clinic staff mentioned that they typically do not provide parent only sessions while the adolescent is in care. It is important to consider whether facilities will be able to integrate this type of service into their regular practice (e.g., is it a billable expense). However, the evidence gained in this study may be what is needed to make this kind of shift in practice.

1. Significance:

Strengths

- This study seeks to evaluate a scalable program to reduce substance use in a high-risk population, adolescents in residential substance use treatment (major)
- Parents are key to preventing relapse for adolescents who require substance use treatment, yet they are difficult to engage in treatment (major)
- A challenge is that 25% of downloaded apps are only used once, research is needed on continued engagement (moderate)
- Existing interventions are time-intensive and create feasibility challenges for staff. Technology assisted interventions can serve as “clinician extenders” to enhance reach. They can reduce burden on frontline staff, limited financial resources, and supervisory support. They also provide some degree of anonymity for families, which is important in addressing sensitive topics (major)

- This proposal builds on a previous NIDA-funded pilot and formative work with parents, adolescents, and treatment staff. The intervention, which includes an app and up to four tele-coaching sessions, has evidence of feasibility, acceptability, parent satisfaction, and preliminary effectiveness (monitoring, communication, binge drinking, drug use, and school problems). This proposal is responsive to parent and staff feedback. For example, program starts when adolescents are in patient, a point when parents have more time to engage. Based on pilot work showing better effects in a short-term compared to longer term residential programs, the decision was made to focus on short-term (major)

Weaknesses

- Clinic staff noted it is rare to have parent only sessions while inpatient. It is not clear whether this could be integrated into the clinic practice (e.g., is it a billable expense) (moderate)
- The research team will serve as moderators of the networking app, which compromises the external validity of the study. It is not clear if this could be scaled up by clinic staff with fidelity. (moderate)
- There may be a gap between when parents initiative the program and when they actually have an opportunity to practice the skills with adolescents, however, given short-term residential programs are targeted, this gap is unlikely to be long (minor)

2. Investigator(s):

Strengths

- Investigators have conducted formative research guiding the development of the program and initial feasibility testing. (major)
- Th PI has conducted reviews and meta-analyses that demonstrate the importance of including parents in interventions for adolescent substance use outperformed treatments that did not include parents (moderate)
- The team has previously conducted studies comparing engagement in technology-based interventions, which provided justification for the inclusion of the telehealth sessions (major)
- The team has a history of collaboration with the clinical sites (major)
- The PI and Co-Is have a history of NIDA and NIMH funded clinical studies with high-risk adolescents. (major)
- The team also has methodological expertise relevant to the study, including behavioral observation, longitudinal, and multi-method designs. (major)

Weaknesses

- None noted by reviewer.

3. Innovation:

Strengths

- This proposal is innovative in delivering a parenting intervention to prevent relapse for adolescents in residential treatment. The timing of the intervention (starting while adolescent is in care) is particularly innovative and likely to be impactful, given that it occurs at a time when parents may be most motivated to make changes, and able to engage in the intervention, given the reductions in their parenting responsibilities. (major)

- Hybrid approach with off the shelf intervention paired with telehealth sessions and networking site where families can connect with a therapist of other parents (major)
- Testing of mediation: this is not a new approach, but still underutilized (moderate)

Weaknesses

- None noted by reviewer.

4. Approach:

Strengths

- Intervention has been piloted for feasibility, acceptability, and preliminary effectiveness; modifications were made based on feedback from parents and staff in a short-term (6-10 days) and long-term (30-45 days) residential programs. Adherence and competence as assessed, and thresholds were reached. (major)
- Evidence-informed recruitment and retention procedures have been developed and piloted. Multiple methods are in place to recruit and retain racial/ethnic minority youth. All staff will be bilingual/bicultural. All staff will be trained in culturally competent service delivery. The program materials are already available in Spanish (major)
- Fidelity monitoring (adherence and competent delivery) procedures have been piloted. (major)
- Multi-method assessment battery with validated measures: parent and adolescent self-report, interaction task, urine screen. (major)
- Four data collection points, but only up until 24-weeks post-discharge (moderate)
- Will use mediational modeling to test theoretical pathways from changes in parenting to changes in adolescent SU outcomes (moderate)
- An answer bank has already been established for the “ask an expert” forum (moderate)
- Proposed statistical analyses are appropriate and the study is sufficiently powered for the primary and secondary aims. (major)

Weaknesses

- It is not explained how thresholds for adherence and competence were achieved. It is not stated what will occur if counselors do not achieve the threshold on the roleplay. There also does not appear to be any active training (they will review the manual, workbook, and modules). (moderate)
- Members of the investigative team are responsible for all aspects of the networking app. This causes concerns about external validity and sustainability. Specifically, if the program is deemed effective, it is unknown whether an agency could replicate these effects. It is also important to consider if there are staff available who could implement this piece and how they would be compensated for their time. (major)
- A primary rationale for the study is on engagement, however, the protocol does not mention an assessment of engagement in the various components of the intervention. (moderate)

5. Environment:

Strengths

- Brown University's Center for Alcohol and Addictions Studies (CAAS) is an internationally renowned research center in substance use and addictions research
- CAAS is home to New England Addiction Technology Center (ATTC), led by PI Becker, a SAMHSA-funded regional center tasked with training substance use treatment providers across the 6-state New England region.
- Resources available for the project appear to be sufficient at the university and clinical sites.

Weaknesses

- None noted by reviewer.

Study Timeline:

Strengths

- Timeline is clear and appears feasible

Weaknesses

- None noted by reviewer.

Protections for Human Subjects:

Acceptable Risks and/or Adequate Protections

- The study includes a vulnerable population (adolescents in residential treatment). The study team has clinical training and experience in working with this population. Study procedures are in place to mitigate risk

Data and Safety Monitoring Plan (Applicable for Clinical Trials Only):

Acceptable

- The PI has responsibility for developing and executing the DSMP. The DSMB will be chaired by a licensed clinical psychologist and NIH-funded researcher with experience in clinical trials. Other members will be drawn from the Center for Alcohol and Addictions Studies DSMB group and will include clinical and quantitative methods expertise. Meetings will be held twice a year and additionally as needed. The PI will draft a report prior to each meeting for review. AE and SAEs will be reported to the DSMB and IRB within 72 hours. SAEs will also be reported to NIDA. The DSMB will issue recommendations that the PI will be responsible for addressing. This information will be included in the annual report to NIDA, which the DSMB will review.

Inclusion Plans:

- Sex/Gender: Distribution justified scientifically
- Race/Ethnicity: Distribution justified scientifically
- For NIH-Defined Phase III trials, Plans for valid design and analysis: Not applicable
- Inclusion/Exclusion Based on Age: Distribution justified scientifically
- No participants will be excluded on the basis of sex, race, or ethnicity. The intervention is designed to improve outcomes for adolescents in residential treatment setting (13-18 years) by intervening with parents (no age restrictions)

Vertebrate Animals:

Not Applicable (No Vertebrate Animals)

Biohazards:
Not Applicable (No Biohazards)

Resource Sharing Plans:
Acceptable

Budget and Period of Support:
Recommend as Requested

CRITIQUE 2

Significance: 2
Investigator(s): 1
Innovation: 2
Approach: 2
Environment: 1

Overall Impact: This R01 clinical trial application from Sara Becker proposes to build on her successful track record of research on adolescent and parent interventions to test Parent SMART, a technology-assisted enhancement of the evidence-based Parenting Wisely program, augmented for parents of teens who are completing residential treatment for substance use. The team is exceptional. The work stems directly from PI Becker's NIDA-funded R34, which yielded rich pilot data that guided the design of the proposed study in some very specific ways. The study is significant because adolescents in residential treatment have high rates of relapse, and there is good evidence that involving parents in interventions improves outcomes. The approach is generally rigorous and well thought-out, with design decisions informed by prior evidence from the literature and the team's research, with relevant alternatives thoroughly considered. The study design and execution plan includes various elements to enhance methodological rigor. Weaknesses are generally minor and do not detract from enthusiasm for this excellent application.

1. Significance:
Strengths
- Adolescents in residential treatment are at high risk of relapse; strengthening parents' communication and monitoring capacity could improve outcomes in this vulnerable group.
- The application provides a thorough review of the prior research on the role of parent-youth communication and parental monitoring, the literature on the Parenting Wisely intervention, and the technology-assisted interventions. This review suggests that the proposed intervention builds upon a rigorous body of prior research, and the application articulates clearly how the intervention advances the current state of the science.

Weaknesses
- Adolescents undergoing short-term residential treatment for SUDs, with parents who are willing and able to engage in an extended technology-based intervention after treatment, are a fairly narrow (although certainly high risk) population.

2. Investigator(s):

Strengths

- Strong group of investigators led by PI Becker, an experienced PI and expert in adolescent and parenting interventions with a strong track record of scientific contributions.
- Co-I Spirito brings senior level expertise in adolescent clinical trials
- Co-I Helseth is an early career scientist with an growing record of scientific contributions, working closely with the PI and Co-Is
- Other Co-Is are likewise strong. The team has a history of successful collaboration.

Weaknesses

- None noted.

3. Innovation:

Strengths

- Parent Smart includes innovative enhancements to an evidence-based intervention (Parenting Wisely), including in-person telehealth support, expert-moderated networking, a parenting forum, and push notifications. Taken together, these features are a novel and meaningful enhancement to an existing intervention.
- The intervention is novel in allowing parents to connect with experts, other parents, and automated content.

Weaknesses

- None noted

4. Approach:

Strengths

- Preliminary studies are strong, relevant, and have informed the design in specific ways. It is clear that the investigators learned a great deal from the R34 experience and incorporated concrete refinements into the intervention based on parent feedback (e.g., timing of sessions). Likewise, the pilot findings informed fundamental design features of the proposed trial, such as focusing the targeted population on short- rather than long-term residential treatment. In general, the preliminary findings support moving towards a full-scale RCT.
- Well-considered casting of the study towards the effectiveness side of the efficacy-effectiveness spectrum, while retaining elements to strengthen internal validity.
- Strong RCT design.
- TAU as the comparator arm is reasonable and will inform practice.
- Proximal outcomes for parental monitoring and communication will be considered, as well as adolescent substance use outcomes and problem behaviors.
- Trial includes rigorous plans for fidelity monitoring.
- Residential treatment staff, assessors, and analyst will be blind to assignment (rigor).
- Measures are well-validated, and include adolescent drug testing biomarkers.

- The intervention is available in English and Spanish, broadening reach.
- Recruitment projections are well-justified based on treatment admission flow and pilot data. Recruitment and Retention plan is superb and adds confidence that high follow-up rates will be achieved.
- Proposed analysis methods are sophisticated.

Weaknesses

- The Parent SMART intervention relies on considerable ongoing staff support. The ultimate scalability of this intervention is an open question. Unclear what entity would pay for the infrastructure going forward, although this is a minor concern at this stage (largely a question for implementation science after effectiveness is established).
- Follow-up of only 24 weeks in duration will not capture longer-term effects (minor).
- Unclear if the testing panel will include fentanyl (minor).
- Rationale for including 18 year olds is unclear. It is possible that adults could be very different from minors with respect to relationships with parents and autonomy (minor).
- Although power appears adequate to detect small-moderate effect sizes on the intermediate outcomes of parenting dynamics and communication, with N=220 the study may be underpowered to detect effects on the ultimate target of adolescent relapse. (minor)

5. Environment:

Strengths

- Brown University is an outstanding environment with ample resources and infrastructure to support the study.
- Recruitment sites ART at McLean hospital and the Bradley Center are excellent clinical sites for recruiting the target population of adolescent-parent dyads.

Weaknesses

- None noted

Study Timeline:

Strengths

- The study timeline is reasonable, calibrated to key study activities and the flow of adolescents at the recruitment sites.

Weaknesses

- None noted.

Protections for Human Subjects:

Acceptable Risks and/or Adequate Protections

- No concerns. The application considers risks in considerable detail and articulates plans to minimize them.

Data and Safety Monitoring Plan (Applicable for Clinical Trials Only):

Acceptable

- No concerns. The study will be monitored by a DSMB.

Inclusion Plans:

- Sex/Gender: Distribution justified scientifically
- Race/Ethnicity: Distribution justified scientifically
- For NIH-Defined Phase III trials, Plans for valid design and analysis: Not applicable
- Inclusion/Exclusion Based on Age: Distribution justified scientifically
- Adolescent and parent dyads; adolescents ages 13-18, scientifically justified. Adolescents will be 65% white and 50% female. Parents are expected to be 80% female.

Vertebrate Animals:

Not Applicable (No Vertebrate Animals)

Biohazards:

Not Applicable (No Biohazards)

Resource Sharing Plans:

Not Applicable (No Relevant Resources)

Budget and Period of Support:

Recommend as Requested

CRITIQUE 3

Significance: 1
Investigator(s): 1
Innovation: 2
Approach: 3
Environment: 1

Overall Impact: This new R01 application by Dr. Sara Becker (PI) will enroll 220 adolescent/parent dyads in short-term residential substance use (SU) treatment. This two-group RCT is designed to evaluate the effectiveness of a technology-assisted parenting intervention called Parent SMART (Substance Misuse among Adolescents in Residential Treatment) + TAU versus TAU on parent outcomes: parental monitoring and communication and adolescent substance use outcomes: days of use and substance-related problems (primary outcomes) and adolescent problem behavior (secondary outcomes). They will also evaluate the extent to which change in parenting processes mediates change in adolescent substance outcomes. Parent SMART was built from a known computer program called Parenting Wisely (PW) which has shown effectiveness in improving parenting skills and youth behavior problems. PW was augmented in Parent SMART to include: a) access to an expert-moderated mobile networking forum with push notifications and SMS messages and b) up to 4 telehealth sessions to tailor delivery of the PW content. The sessions begin during the teen's residential treatment. Outcomes will be measured at 6-, 12-, and 24-weeks after discharge. This research area is significant and the rigor is

high as Parent SMART has been tested in both short-and long-term residential facilities in a NIDA-funded R34 which demonstrated high feasibility, acceptability in both settings and preliminary effectiveness. Results showed significant improvements in parental monitoring and communication, reduced adolescent: SU days (reductions in binge drinking, all other drug use) and reduced school-related problems in families receiving short-term residential treatment. The research team is outstanding. The timing of delivery, the elements included (video, telehealth), and the testing of mediators of treatment effects of Parent SMART are innovative. The approach is rigorous including the measures used, plan for randomization, statistical analyses, and measures of SU with urine tests. Some concerns in the approach include the length of assessments, the inclusion of parents who complete all available counseling sessions, and the self-reported measure of alcohol use. Overall, this project is needed in this high-risk population, it is rigorous as it is backed up by strong pilot data, and it is likely to be effective in achieving planned outcomes.

1. Significance:

Strengths

- This research is significant as adolescents in residential SU treatment have the highest rates of SU disorders and high rates of related psychological, behavioral and other sequelae. Rates of relapse within 90 days after discharge from residential SU treatment is as high as 60%.
- The rigor is high as Parent SMART has been tested in both short-and long-term residential facilities in a NIDA-funded R34 which demonstrated high feasibility, acceptability in both settings and preliminary effectiveness. Results showed significant improvements in parental monitoring and communication, reduced adolescent: SU days (reductions in binge drinking, all other drug use) and school-related problems in families receiving short-term residential treatment.

Weaknesses

- None noted.

2. Investigator(s):

Strengths

- The research team is outstanding. Dr. Becker (PI) has published three systematic reviews and meta-analyses of treatment approaches for adolescent SU which found that parent-based SU interventions are more effective in achieving SU treatment outcomes compared to adolescent-only approaches. She has clinical experience at the McLean ART program and she has served on relevant advisory boards.
- Dr. Spirito (Co-I) and Dr. Becker have conducted clinical research at the Bradley Center since 2009.
- Dr. Helseth (Co-I) is an expert in the development and testing of technology-based interventions for high-risk youth and she has trained Parent SMART coaches.
- The study team has worked well together and each member has complementary expertise that will add to this project.

Weaknesses

- None noted.

3. Innovation:

Strengths

- The timing of delivery and the elements included (video, telehealth) in the intervention are innovative
- The testing of mediators of treatment effects of Parent SMART is innovative.

Weaknesses

- The use of mobile apps as interventions is not that innovative in general.

4. Approach:

Strengths

- The plan for urn randomization by sex, treatment site and preferred language is sound. Residential treatment staff are blind to condition.
- 25% of the counseling sessions will be jointly reviewed and 20% will be double coded.
- Based on the R34, a repository of over 150 questions and comments and an answer bank has already been created. This will make it easier to respond to questions from participants in a timely fashion.
- There is high scientific rigor: measures have high reliability (alphas >0.7), the statistical analyses and sample size calculations are rigorous.
- The measures used are validated with good reliability. The plan for statistical analysis is sound.
- Urine screens will be collected to validate MJ, cocaine, amphetamines, methamphetamines, barbiturates, PCP, opiates, benzodiazepines.
- Very detailed retention strategies are presented which will likely result in >80% retention rates. Incentives are generous which will encourage participation and retention.
- Outcomes are assessed at 6, 12, and 24-weeks.

Weaknesses

- Although mediators of the intervention effects will be examined, there is no conceptual model guiding the study beyond the theory of the social ecology of human development which gives credence to a parent intervention. This is a minor concern since Figure 3 details a conceptual framework that ties the aims and hypotheses.
- Alcohol use will be assessed via self-report.
- The R34 showed greater reductions in MJ in TAU youth which is partially explained. The other SU outcomes were very positive.
- The assessments are very long (adolescents/parents at baseline: 90-120 minutes/45-60 minutes and 60- and 30-minutes follow-up). It may be difficult to get complete compliance of all questions at each timepoint.
- Both participants who complete all sessions or some sessions will be included. Although this will be considered in statistical analysis, the results will likely be very different in the “completers”.
- It is not specified what % of the assessments need to be completed or how many of the 3 urine tests need to be obtained in order for data analysis to be conducted.

5. Environment:

Strengths

- Research activities will be based at Brown University’s Center for Alcohol and Addictions Studies (CAAS). CAAS is home to New England Addiction Technology Center (ATTC) led by the PI, Dr. Becker. This infrastructure and support for this project is very strong.

- Dr. Becker will be able to use the resources and relationships she has established to disseminate study results. These include surrounding treatment centers and hospitals (Bradley Hospital and McLean Hospital).
- There is a strong LOS from Silent Disclosure, the company that will collect and compile metadata to enable analysis of Parent SMART usage and provide ongoing programmatic support. Except for phone numbers and email addresses, no PHI will be accessed by this company and they state they have no COI on this project.

Weaknesses

- None noted.

Study Timeline:

Strengths

- There is a detailed timeline with all study tasks clearly outlined. The proposed dates of accomplishing each task seem feasible. The timeline for recruitment of 220 dyads over 50 months is reasonable.

Weaknesses

- None noted.

Protections for Human Subjects:

Acceptable Risks and/or Adequate Protections

- No human subject concerns

Data and Safety Monitoring Plan (Applicable for Clinical Trials Only):

Acceptable

- DSMP and DSMB are planned
- There are plans for how to address safety concerns

Inclusion Plans:

- Sex/Gender: Distribution justified scientifically
- Race/Ethnicity: Distribution justified scientifically
- For NIH-Defined Phase III trials, Plans for valid design and analysis: Not applicable
- Inclusion/Exclusion Based on Age: Distribution justified scientifically
- Inclusions are all scientifically justified: Both genders, Minority and non-minority. Both Children and adults included- all scientifically justified

Vertebrate Animals:

Not Applicable (No Vertebrate Animals)

Biohazards:

Not Applicable (No Biohazards)

Resource Sharing Plans:

Acceptable

Budget and Period of Support:

Recommend as Requested

THE FOLLOWING SECTIONS WERE PREPARED BY THE SCIENTIFIC REVIEW OFFICER TO SUMMARIZE THE OUTCOME OF DISCUSSIONS OF THE REVIEW COMMITTEE, OR REVIEWERS' WRITTEN CRITIQUES, ON THE FOLLOWING ISSUES:

PROTECTION OF HUMAN SUBJECTS: ACCEPTABLE

INCLUSION OF WOMEN PLAN: ACCEPTABLE

INCLUSION OF MINORITIES PLAN: ACCEPTABLE

INCLUSION ACROSS THE LIFESPAN: ACCEPTABLE

COMMITTEE BUDGET RECOMMENDATIONS: The budget was recommended as requested.

Footnotes for 1 R01 DA052918-01; PI Name: Becker, Sara J.

NIH has modified its policy regarding the receipt of resubmissions (amended applications).See Guide Notice NOT-OD-18-197 at https://grants.nih.gov/grants/guide/notice-files/NOT-OD-18-197.html. The impact/priority score is calculated after discussion of an application by averaging the overall scores (1-9) given by all voting reviewers on the committee and multiplying by 10. The criterion scores are submitted prior to the meeting by the individual reviewers assigned to an application, and are not discussed specifically at the review meeting or calculated into the overall impact score. Some applications also receive a percentile ranking. For details on the review process, see http://grants.nih.gov/grants/peer_review_process.htm#scoring.

MEETING ROSTER

Interventions to Prevent and Treat Addictions Study Section
Risk, Prevention and Health Behavior Integrated Review Group
CENTER FOR SCIENTIFIC REVIEW
IPTA
06/04/2020 - 06/05/2020

Notice of NIH Policy to All Applicants: Meeting rosters are provided for information purposes only. Applicant investigators and institutional officials must not communicate directly with study section members about an application before or after the review. Failure to observe this policy will create a serious breach of integrity in the peer review process, and may lead to actions outlined in NOT-OD-14-073 at https://grants.nih.gov/grants/guide/notice-files/NOT-OD-14-073.html and NOT-OD-15-106 at https://grants.nih.gov/grants/guide/notice-files/NOT-OD-15-106.html, including removal of the application from immediate review.

CHAIRPERSON(S)

WALTON, MAUREEN A, PHD, MPH
PROFESSOR
DEPARTMENT OF PSYCHIATRY
UNIVERSITY OF MICHIGAN
ANN ARBOR, MI 48109

MEMBERS

ALESSI, SHEILA MARIE, PHD *
ASSOCIATE PROFESSOR
DEPARTMENT OF PSYCHIATRY
CALHOUN CARDIOLOGY CENTER
UNIVERSITY OF CONNECTICUT HEALTH CENTER
FARMINGTON 06030

BERKEL, CADY, PHD *
ASSOCIATE RESEARCH PROFESSOR
REACH INSTITUTE
SOUTHWEST INTERDISCIPLINARY RESEARCH CENTER
ARIZONA STATE UNIVERSITY
TEMPE, AZ 85284

BERMAN, MITCHELL E, PHD *
PROFESSOR AND DEPARTMENT HEAD
DEPARTMENT OF PSYCHOLOGY
MISSISSIPPI STATE UNIVERSITY
MISSISSIPPI STATE, MS 39762

BERNSTEIN, STEVEN L, MD
PROFESSOR AND VICE CHAIR
DEPARTMENT OF EMERGENCY MEDICINE
YALE UNIVERSITY SCHOOL OF MEDICINE
NEW HAVEN, CT 06519

BRADIZZA, CLARA M, PHD
PROFESSOR
SCHOOL OF SOCIAL WORK
UNIVERSITY AT BUFFALO
STATE UNIVERSITY OF NEW YORK
BUFFALO, NY 14203

BUCKNER, JULIA D, PHD *
PROFESSOR AND DIRECTOR OF CLINICAL TRAINING
DEPARTMENT OF PSYCHOLOGY
LOUISIANA STATE UNIVERSITY
BATON ROUGE, LA 70803

COLEMAN-COWGER, VICTORIA HOPE, PHD
CLINICAL RESEARCH DIRECTOR
THE EMMES CORPORATION
ROCKVILLE, MD 20850

CROPSEY, KAREN L, PSYD
PROFESSOR
DEPARTMENT OF PSYCHIATRY AND BEHAVIORAL NEUROLOGY
UNIVERSITY OF ALABAMA AT BIRMINGHAM
BIRMINGHAM, AL 35294

DUNN, KELLY E., PHD *
ASSOCIATE PROFESSOR
DEPARTMENT OF PSYCHIATRY AND BEHAVIORAL SCIENCES
SCHOOL OF MEDICINE
JOHNS HOPKINS UNIVERSITY
BALTIMORE, MD 21224-6823

FRIEDMANN, PETER D, MD, MPH
PROFESSOR
DEPARTMENT OF MEDICINE
UNIVERSITY OF MASSACHUSETTS
MEDICAL SCHOOL - BAYSTATE
SPRINGFIELD, MA 01107

GRANT, SEAN, PHD *
ASSISTANT PROFESSOR
RICHARD M. FAIRBANKS SCHOOL OF PUBLIC HEALTH
INDIANA UNIV-PURDUE UNIV AT INDIANAPOLIS
INDIANAPOLIS, IN 46202

GRYCZYNSKI, JAN, PHD
SENIOR RESEARCH SCIENTIST
FRIENDS RESEARCH INSTITUTE
BALTIMORE, MD 21201

HETTEMA, JENNIFER ELIN, PHD *
ASSOCIATE PROFESSOR
DEPARTMENT OF FAMILY
AND COMMUNITY MEDICINE
UNIVERSITY OF NEW MEXICO SCHOOL OF MEDICINE
ALBUQUERQUE 87131

HILL, KEVIN P., MD *
ASSOCIATE PROFESSOR OF PSYCHIATRY
HARVARD MEDICAL SCHOOL
BOSTON, MA 02215

HITSMAN, BRIAN L, PHD
ASSOCIATE PROFESSOR
DEPARTMENT OF PREVENTIVE MEDICINE
FEINBERG SCHOOL OF MEDICINE
NORTHWESTERN UNIVERSITY
CHICAGO, IL 60611

INGERSOLL, KAREN S, PHD *
PROFESSOR
DEPARTMENT OF PSYCHIATRY
AND NEUROBEHAVIORAL SCIENCES
UNIVERSITY OF VIRGINIA
CHARLOTTESVILLE, VA 22911

KAHLER, CHRISTOPHER W., PHD *
PROFESSOR AND CHAIR
DEPARTMENT OF BEHAVIORAL
AND SOCIAL SCIENCES
CENTER FOR ALCOHOL AND ADDICTION STUDIES
BROWN UNIVERSITY SCHOOL OF PUBLIC HEALTH
PROVIDENCE, RI 02912

KIM-MOZELESKI, JIN E., PHD *
ASSISTANT PROFESSOR
DEPARTMENT OF POPULATION AND
QUANTITATIVE HEALTH SCIENCES
CASE WESTERN RESERVE UNIVERSITY
CLEVELAND, OH 44106

LARSON, MARY JO, PHD
SENIOR SCIENTIST
INSTITUTE FOR BEHAVIORAL HEALTH
HELLER SCHOOL FOR SOCIAL POLICY AND MANAGEMENT
BRANDEIS UNIVERSITY
WALTHAM, MA 02454

LITT, DANA M, PHD *
ASSOCIATE PROFESSOR
DEPARTMENT OF HEALTH BEHAVIOR AND HEALTH
SYSTEMS
SCHOOL OF PUBLIC HEALTH SYSTEMS
UNIVERSITY OF NORTH TEXAS
FORT WORTH 76107

LOVEJOY, TRAVIS IAN, MPH, PHD *
ASSOCIATE PROFESSOR
DEPARTMENT OF PSYCHIATRY
SCHOOL OF MEDICINE
OREGON HEALTH AND SCIENCE UNIVERSITY
PORTLAND, OR 97239

MAHABEE-GITTENS, E. MELINDA, MD
PROFESSOR
DIVISION OF EMERGENCY MEDICINE
CINCINNATI CHILDREN'S HOSPITAL MEDICAL CENTER
CINCINNATI, OH 45229

MAISTO, STEPHEN A, PHD *
PROFESSOR
DEPARTMENT OF PSYCHOLOGY
SYRACUSE UNIVERSITY
SYRACUSE, NY 13244

MCGOVERN, MARK P, PHD
PROFESSOR
DEPARTMENT OF PSYCHIATRY
AND BEHAVIORAL SCIENCES
STANFORD UNIVERSITY
PALO ALTO, CA 93404

MENDELSON, TAMAR, PHD
PROFESSOR
DEPARTMENT OF MENTAL HEALTH
JOHNS HOPKINS BLOOMBERG SCHOOL OF PUBLIC HEALTH
BALTIMORE, MD 21205

MILLER, MARY ELIZABETH, PHD *
ASSISTANT PROFESSOR
DEPARTMENT OF PSYCHIATRY
UNIVERSITY OF MISSOURI
COLUMBIA, MO 65211

MONTGOMERY, LATRICE, PHD *
ASSISTANT PROFESSOR
ADDICTION SCIENCES DIVISION
DEPARTMENT OF PSYCHIATRY
AND BEHAVIORAL NEUROSCIENCE
UNIVERSITY OF CINCINNATI COLLEGE OF MEDICINE
CINCINNATI, OH 45229

NOONAN, DEVON, PHD *
ASSOCIATE PROFESSOR
SCHOOL OF NURSING
DUKE UNIVERSITY
DURHAM, NC 27710

OLIVETO, ALISON, PHD
PROFESSOR AND VICE CHAIR FOR RESEARCH
DEPARTMENT OF PSYCHIATRY
UNIVERSITY OF ARKANSAS FOR MEDICAL SCIENCES
LITTLE ROCK, AR 72205

OSILLA, KAREN C, PHD
SENIOR BEHAVIORAL SCIENTIST
RAND CORPORATION
SANTA MONICA, CA 90404

PARROTT, DOMINIC, PHD *
PROFESSOR
DEPARTMENT OF PSYCHOLOGY
GEORGIA STATE UNIVERSITY
ATLANTA, GA 30302

PIPER, MEGAN E, PHD
ASSOCIATE PROFESSOR
DEPARTMENT OF MEDICINE
UNIVERSITY OF WISCONSIN-MADISON
MADISON, WI 53711

SCHEUERMANN, TANEISHA SHANI, PHD *
ASSISTANT PROFESSOR
DEPARTMENT OF PREVENTIVE MEDICINE AND
PUBLIC HEALTH
UNIVERSITY OF KANSAS MEDICAL CENTER
KANSAS CITY, KS 66160

STEIN, MICHAEL D, MD
PROFESSOR AND CHAIR
DEPARTMENT OF HEALTH LAW, POLICY AND MANAGEMENT
BOSTON UNIVERSITY SCHOOL OF PUBLIC HEALTH
BOSTON, MA 02118

STEINBERG, MARC L, PHD *
ASSOCIATE PROFESSOR
DIVISION OF ADDICTION PSYCHIATRY
ROBERT WOOD JOHNSON MEDICAL SCHOOL
RUTGERS, THE STATE UNIVERSITY OF NEW JERSEY
NEW BRUNSWICK, NJ 08901

TINDLE, HILARY A, MD, MPH
ASSOCIATE PROFESSOR
DIVISION OF INTERNAL MEDICINE AND PUBLIC HEALTH
VANDERBILT UNIVERSITY MEDICAL CENTER
NASHVILLE, TN 37203

TSOH, JANICE Y, PHD
PROFESSOR
DEPARTMENT OF PSYCHIATRY
LANGLEY PORTER PSYCHIATRIC INSTITUTE
UNIVERSITY OF CALIFORNIA SAN FRANCISCO
SAN FRANCISCO, CA 94143

TSUI, JUDITH, MD, MPH *
ASSOCIATE PROFESSOR OF MEDICINE
DIVISION OF GENERAL INTERNAL MEDICINE
UNIVERSITY OF WASHINGTON SCHOOL OF MEDICINE
HARBORVIEW MEDICAL CENTER
SEATTLE, WA 98122

VELASQUEZ, MARY M, PHD
CENTENNIAL PROFESSOR AND DIRECTOR
HEALTH BEHAVIOR RESEARCH
AND TRAINING INSTITUTE
STEVE HICKS SCHOOL OF SOCIAL WORK
UNIVERSITY OF TEXAS AT AUSTIN
AUSTIN, TX 78712

VINCI, CHRISTINE, PHD *
ASSISTANT MEMBER
DEPARTMENT OF HEALTH OUTCOMES AND BEHAVIOR
MOFFITT CANCER CENTER
TAMPA, FL 33612

WINSTANLEY, ERIN L, PHD *
ASSOCIATE PROFESSOR
DEPARTMENT OF BEHAVIORAL MEDICINE AND PSYCHIATRY
WEST VIRGINIA UNIVERSITY, SCHOOL OF MEDICINE
MORGANTOWN, WV 26506

YI, RICHARD, PHD *
PROFESSOR
DEPARTMENT OF PSYCHOLOGY
COFRIN LOGAN CENTER FOR ADDICTION RESEARCH AND
TREATMENT
UNIVERSITY OF KANSAS
LAWRENCE, KS 66045

SCIENTIFIC REVIEW OFFICER

MINTZER, MIRIAM, PHD
SCIENTIFIC REVIEW OFFICER
CENTER FOR SCIENTIFIC REVIEW
NATIONAL INSTITUTES OF HEALTH
BETHESDA, MD 20892

EXTRAMURAL SUPPORT ASSISTANT

FAYEMIWO, TOLU, MS
EXTRAMURAL SUPPORT ASSISTANT
CENTER FOR SCIENTIFIC REVIIEW
NATIONAL INSTITUTES OF HEALTH
BETHESDA, MD 20892

* Temporary Member. For grant applications, temporary members may participate in the entire meeting or may review only selected applications as needed.

Consultants are required to absent themselves from the room during the review of any application if their presence would constitute or appear to constitute a conflict of interest.
